# Proteomic Analysis of the Amygdala Reveals Dynamic Changes in Glutamate Transporter-1 During Progression of Complete Freund’s Adjuvant-Induced Pain Aversion

**DOI:** 10.1007/s12035-023-03415-7

**Published:** 2023-08-04

**Authors:** Yuanyuan Wu, Yuerong Chen, Yunyun Xu, Wenqin Ni, Chalian Lin, Xiaomei Shao, Zui Shen, Xiaofen He, Chao Wang, Jianqiao Fang

**Affiliations:** 1 Key Laboratory of Acupuncture and Neurology of Zhejiang Province, Department of Neurobiology and Acupuncture Research, The Third Clinical Medical College, Zhejiang Chinese Medical University, Hangzhou, China; 2 The Third Clinical Medical College, Zhejiang Chinese Medical University, Hangzhou, Zhejiang China

**Keywords:** Pain aversion,, Amygdala,, Proteomics,, Glutamate transporter-1, Glutamatergic neuron

## Abstract

Pain sufferer usually show an aversion to the environment associated with pain, identified as pain aversion. The amygdala, an almond-shaped limbic structure in the medial temporal lobe, exerts a critical effect on emotion and pain formation. However, studies on inflammatory pain-induced aversion are still relatively limited, and the available evidence is not enough to clarify its inherent mechanisms. Proteomics is a high-throughput, comprehensive, and objective study method that compares the similarities and differences of protein expression under different conditions to screen potential targets. The current study aimed to identify potential pivotal proteins in the amygdala of rats after complete Freund’s adjuvant (CFA)-induced pain aversion via proteomics analysis. Immunohistochemistry was performed to confirm the expression of glutamate transporter-1 (GLT-1) in the amygdala during different periods of pain aversion. Thirteen proteins were found to be different between the day 2 and day 15 groups. Among the 13 differentially expressed proteins, Q8R64 denotes GLT-1, which utilises synaptic glutamate to remain optimal extracellular glutamic levels, thereby preventing accumulation in the synaptic cleft and consequent excitotoxicity. The variation in GLT-1 expression was correlated with the variation tendency of pain aversion, which implies a potential link between the modulation of pain aversion and the excitability of glutamatergic neurons. This study demonstrated that exposure to inflammatory pain results in aversion induced from pain, leading to extensive biological changes in the amygdala.

**Supplementary Information:**

The online version contains supplementary material available at 10.1007/s12035-023-03415-7.

## Introduction

Patients having pain episodes usually tend to avoid pain-associated environments, defined as pain aversion. Previous research has revealed that physical pain and emotions can reciprocally affect each other. Negative emotions such as anxiety and depression enhance the risk of chronic pain, which can in turn result in further depression and anxiety. For instance, studies have suggested that the risk of developing chronic musculoskeletal pain is two-fold higher in patients with depression compared to that in healthy controls [1], and the severity of depressive symptoms in patients is correlated with the likelihood of developing neck and back pain [2, 3].

Emotional responses originate from the activation of specialised neuronal populations in several parts of the cerebral cortex, notably the ventromedial prefrontal, insula, anterior cingulate, and subcortical structures, such as the amygdala, nucleus accumbens, and ventral striatum [4–8]. The amygdala, located in the medial temporal lobe with an almond-shaped limbic structure, is well known for its role in assigning emotional significance to sensory inputs, affective and emotional states, and related behavioural adaptations [9–13].

Recently, the amygdala has received growing attention due to its role in the modulation of pain perception [14, 15]. Indeed, widespread mechanical hypersensitivity in chronic back pain model can be debilitated by inhibition of GABAergic neurons in the right amygdala [16]. Structural changes of synapses in the amygdala impair the glutamate delta-1 receptor and its partner cerebellin-1 in inflammatory and neuropathic pain settings [17]. Also, a pain state may produce physiological alterations in opioid transmission in the amygdala [18]. Moreover, animal research has shown that TNF-α promotes the exacerbation of anxiety by maintaining certain pain levels [19]. However, studies on pain aversion are still relatively rare. The available evidence is far from sufficient to clarify its underlying mechanisms.

Isobaric tags for relative and absolute quantitation (iTRAQ) are used for quantitative proteomics performed by tandem mass spectrometry. iTRAQ is applied to identify the quantity of protein from different sources in a single experiment. It uses stable isotopes to label molecules that can covalently bind to the N-terminal and side-chain amines of the proteins. An iTRAQ-based neuroproteomics allows to investigate the functional changes resulting from pain aversion at the molecular level [20, 21].

Here, we hypothesised that functional and structural components of the amygdala are engaged in pain aversion. We investigated whether potential pivotal proteins in rat amygdala were affected upon complete Freund’s adjuvant (CFA)-induced pain aversion via iTRAQ. Synthetic analyses of protein expression were performed to explore the potential molecular mechanisms of pain aversion.

## Materials and Study Methods

### Animals

Male Sprague–Dawley rats (230–270 g, aged 10–12 weeks) were obtained from the Shanghai Laboratory Animal Center, Shanghai, China, and raised in 45 × 50 × 25 cm cages with access to water and food. The animals were raised in groups of five under a 12-h light/dark cycle (light cycle 8:00 AM–8:00 PM) at room temperature (25 ± 1 ℃). Our study was approved by the Laboratory Animal Management and Use Committee of Zhejiang Chinese Medical University. All experiments were approved by the Animal Ethics Committee of Zhejiang Chinese Medical University (ZSLL, 2017–183) and performed in accordance with the guidelines of the National Institutes of Health for the care and use of laboratory animals (NIH Publications No. 8,023, revised 1978).

### Experimental Design

Animals were randomly assigned to the following two groups: (1) saline-injected group (saline, *n* = 9) and (2) CFA-injected group (CFA, *n* = 18), with subgroups including pain aversion days 2 (day 2, *n* = 9) and 15 (day 2, *n* = 9). On day 0, the baseline test of paw withdrawal threshold (PWT) was performed before the saline/CFA injection and was reassessed on days 1 and 14. CFA tests were taken on days 2 and 15.

### CFA-Induced Model and Sham Controls

Inflammatory pain was induced by CFA injection (100 μL) subcutaneously into the plantar surface of the left hind paw. The injection contained 1 mg/mL dried and heat-killed *Mycobacterium tuberculosis* (ATCC 25177) in 0.15 mL of mannide monooleate (Sigma, F5881, USA) and 0.85 mL of paraffin oil. Animals in the saline group were injected with 100 μL of sterile 0.9% saline [22].

### Assessment of Static Mechanical Sensitivity

Prior to the baseline test, the animals were habituated to the testing compartment two to three times. The testing room consisted of clear Plexiglas chambers on a raised wire-mesh grid. Before each test, the rats were housed in the chambers for 30 min for acclimatisation. The assessment of mechanical nociception requires the measurement of 50% of PWT based on Dixon’s study [23].

Mechanical threshold measurements were obtained. Eight von Frey monofilaments (1.4, 2, 4, 6, 8, 10, 15, and 26 g) were used in the following manner. Each test started with a von Frey force weighing 8 g, delivered approximately 5 s to the left hind paw. If there was no paw-flick response, the increased force was applied. In contrast, if there was a reaction, the reduced force was delivered. This program was administered until there was no paw-flick response at the highest force (26 g) or until four stimuli were performed after the initial response. The 50% PWT value was analysed using the following formula: PWT = 10[Xf + kδ], where Xf refers to the value of the final von Frey filament administrated (in log units), k-value represents a figure determined from the pattern of positive/negative responses, and *δ* = 0·184, which is the average interval (in log units) between the eight von Frey filaments. If a rat reacted to the lowest von Frey filament, the value was assigned as 1.4 g. If the rat did not respond to the highest von Frey filament (26 g), a value of 26·0 g was recorded. PWTs were recorded at days 0 (baseline), 1, and 14 after injection.

### Conditioned Place Aversion Paradigm

*The CFA-induced conditioned place aversion (C-CPA) test* was performed as described in a previous report using a place-conditioning device made of Plexiglas [22]. The apparatus consisted of two equal square chambers with 30 cm on each side, positioned on the floor and isolated by guillotine doors. There was no compartment in the centre. One compartment was covered with 23 white equilateral triangles, with a side length of 2.5 cm, and the floor of it was covered with 5% acetic acid. The inner four sides of the other compartment were covered with 30 white dots, 1.8 cm in diameter, at 3.5-cm intervals; the floor of the compartment was covered with cinnamon oil. The area of the compartment with 23 white triangles was identical to that of the compartment with 30 white dots. The triangular spots and coloured dots served as visual cues, while the different substances on the floor served as olfactory cues. Guillotine doors covered with white spots corresponding to their respective sides were fixed during conditioning sessions and dismantled in the process of preconditioning and postconditioning tests. Before each test, the apparatus was cleaned with 75% ethanol. The room was lighted with a 15 W bulb positioned about 1 m above the device.

#### Preconditioning Phase

In the preconditioning phase (day − 1), the baseline time that the rats spent in a 15-min preconditioning time in each of the two different chambers was recorded. The rats were thought in the compartment when the midpoint of the back of rats was inside the compartment.

#### Conditioning Phase

In the conditioning phase (day 1), each rat was allowed to explore one compartment freely for 1 h. For the C-CPA group, all rats were subcutaneously injected with CFA into the plantar surface of the left hind paw (day 1). Two hours after the CFA injection, the animals were allowed to probe another compartment for 1 h. This compartment was referred to as the pain-paired compartment, and the rats were randomly assigned before baseline measurement.

#### Testing Phase

**Fig. 1:**
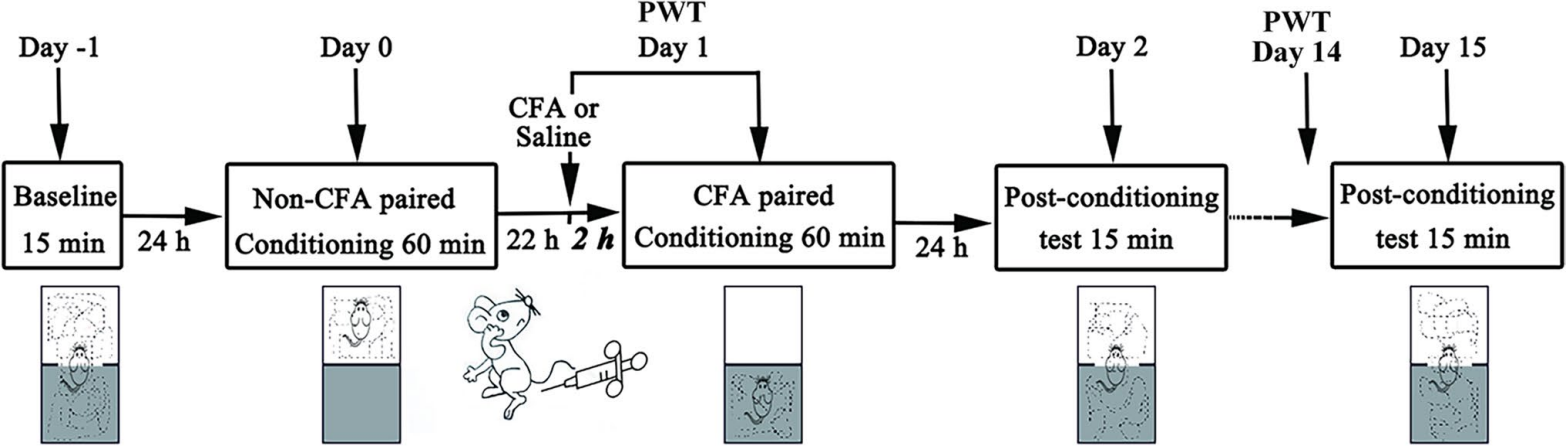
Schematic of the C-CPA timeline. C-CPA, CFA-induced conditioned place aversion; CFA, complete Freund’s adjuvant. Part of the image is redrawn from Wu et al. (2019) [30]

In the process of the 15-min postconditioning phase (day 2), the time that the rats spent in every compartment was also recorded. The CPA score, an implication of affective reaction, was confirmed by subtracting the time spent in the pain-paired compartment in the postconditioning test (day 2) from the time spent in the same chamber in the preconditioning test (day − 1). Less postconditioning time spent in the pain-paired chamber was associated with a stronger affective response. Additional tests were conducted on days 2 and 15. The timeline is shown in Fig. 1.

### Isobaric Tags for Relative and Absolute Quantitation (iTRAQ) Proteomics Analysis

#### Extraction and Digestion of Proteins

The rat brain was quickly removed under deep anaesthesia with 3% sodium pentobarbital (30 mg/kg, i.p.). The right amygdala was obtained and properly stored at temperature of − 80 °C. SDT buffer, contained with 4% SDS, 100 mM Tris–HCl, and 1 mM DTT, was added to each sample. The lysate was homogenised, sonicated, and boiled for about 15 min. After centrifuging the sample at 14,000 × g for 40 min, the supernatant was filtered through 0.22-µm filters, and the BCA Protein Assay Kit (Bio-Rad, USA) was used to quantify the filtrate. Then, 200 μg protein was added into 30 μL SDT buffer. A total of 100 μL iodoacetamide (100 mM IAA in UA buffer) was added to block the reduced cysteine residues, and the samples were incubated for 30 min in the dark. The protein suspensions were digested with 4 μg trypsin (Promega) in 40 μL denaturing SDS (DS) buffer overnight at temperature of 37 °C. The peptides were desalted on C18 Cartridges (Empore SPE Cartridges C18 (standard density), bed I.D. 7 mm, volume 3 mL, Sigma), concentrated by vacuum centrifugation, and reconstituted in 40 μL of 0·1% (v/v) formic acid. The peptide content was calculated through measuring the UV light spectral density at 280 nm using an extinction coefficient of 1.1 of 0.1% (g/l) solution, which was analysed based on the frequency of tryptophan and tyrosine in vertebrate proteins.

#### iTRAQ Labelling and Strong Cation Exchange (SCX) Fractionation

iTRAQ reagent (Applied Biosystems) was applied to label 100 μg of the peptide mixture. iTRAQ-labelled peptides were fractionated by SCX chromatography using an AKTA Purifier system (GE Healthcare). The peptides were eluted at a flow rate of 1 mL/min with a gradient of 0–8% buffer B (500 mM KCl and 10 mM KH_2_PO_4_ in 25% ACN, pH 3·0) for 22 min, followed by 8–52% buffer B during minutes 22–47, 52–100% buffer B during minutes 47–50, 100% buffer B during minutes 50–58, and 0% buffer B after minute 58. Elution was monitored by measuring the absorbance at 214 nm, and fractions were collected every 1 min. The collected fractions were desalted on C18 cartridges (Empore SPE Cartridges C18 (standard density), volume 3 mL, bed I.D. 7 mm, Sigma) and concentrated by vacuum centrifugation.

#### LC–MS/MS Analysis

Each fraction was injected for nano-liquid chromatography coupled with tandem mass spectrometry (LC–MS/MS) analysis. LC–MS/MS analysis was performed using an Easy nLC (Proxeon Biosystems, now Thermo Fisher Scientific) combined with Q Exactive mass spectrometer (Thermo Scientific) for 60 min. MS data were gained using a data-dependent top-10 method, dynamically choosing the most plentiful precursor ions from the survey scan (300–1800 m/z) for HCD fragmentation. The dynamic exclusion duration was 40·0 s. Survey scans were obtained at a resolution of 70,000 at m/z 200, and the resolution for the HCD spectra was determined to be 17,500 at m/z 200 with an isolation width of 2 m/z. The automatic gain control (AGC) target was determined to be 3e6, and the maximum injection time was 10 ms. The normalised collision energy was 30 eV, and the underfill ratio, which specified the minimum percentage of the target value likely to be reached at the maximum fill time, was defined as 0.1%. The instrument was run using the peptide recognition mode.

#### Data Analysis

MS/MS spectra were searched by the MASCOT engine (version 2.2, Matrix Science, London, UK) embedded into Proteome Discoverer 1.4. Setting parameters are shown in Table 1.

**Table 1:** Analysis parameters of MASCOT. This Table is from Wu et al. (2019) [30]

Item	Value
Enzyme	Trypsin
Max missed cleavages	2
Fixed modifications	Carbamidomethyl (C), iTRAQ 4/8plex (N-term), iTRAQ 4/8plex (K)
Variable modifications	Oxidation (M), iTRAQ 4/8plex (Y)
Peptide mass tolerance	± 20 ppm
Fragment mass tolerance	0·1 Da
Peptide FDR	≦0·01
Protein quantification	The protein ratios are calculated as the median of only unique peptides of the protein
Experimental bias	Normalises all peptide ratios by the median protein ratio. The median protein ratio should be 1 after the normalisation

### Bioinformatics Analysis

Kyoto Encyclopaedia of Genes and Genomes (KEGG) pathway enrichment analyses and gene ontology (GO) enrichment on three ontologies, molecular function (MF), cellular component (CC), and biological process (BP), were performed using Fisher’s exact test, with the entire quantified protein annotations as the background dataset. Benjamini–Hochberg correction for multiple testing was further used to rectify the derived *p*-values, and merely functional pathways and categories with *p*-values less than a threshold of 0.05 were considered significant. Hierarchical clustering analysis was performed for relevant protein expression data.

### Immunofluorescence of Glutamate Transporter-1 (GLT-1) in the Amygdala

After the behavioural testing period, the subjects (*n* = 3) in each group were sacrificed with 3% sodium pentobarbital (30 mg/kg, i.p.) and transcardially perfused with about 200 mL pre-cooled 0.9% (w/v) saline followed by 300 mL 4% (w/v) paraformaldehyde solution. Brains of tested rats were then removed and post-fixed in paraformaldehyde overnight for 24 h before storing in 15% (w/v) sucrose solution overnight. The brains were transferred to a 30% (w/v) sucrose solution and stored in a freezer at − 80 ℃ before embedding in the OCT embedding matrix. Cryostat sections at 30 μm around the basolateral amygdala region on a sliding microtome were blocked in 10% donkey serum with 0.3% Triton X-100 for 60 min and then incubated with anti-GLT-1 primary antibody (1:200, CST, 3838S) at 4 ℃ for 20 h. Immunoreactivity to the antigen was visualised using donkey anti-rabbit (Alexa Fluor 488-conjugated) secondary antibodies (1:1000). All images were captured through an Imager M2 microscope (ZEISS, Germany).

### Statistical Analysis

All data are expressed as mean ± standard error of the mean (SEM). Repeated-measures analysis of variance (ANOVA) was used to analyse PWT and CPA scores, followed by Bonferroni’s post hoc tests. For subsequent multiple comparisons, when equal variances were assumed or not assumed, the least significant difference (LSD) and Dunnett’s T3 test were used, as determined by the homogeneity of variance test.

### Data Availability

The datasets generated and/or analysed in the current study are available from the corresponding author.

### Role of the Funding Source

No funding source was involved in the design or implementation of the study; collection, analysis, or interpretation data; or the submission of manuscripts. The corresponding authors are entitled to all data and have the ultimate responsibility for submitting them for publication.

## Results

### CFA Injection Induced Mechanical Hypersensitivity

**Fig. 2:**
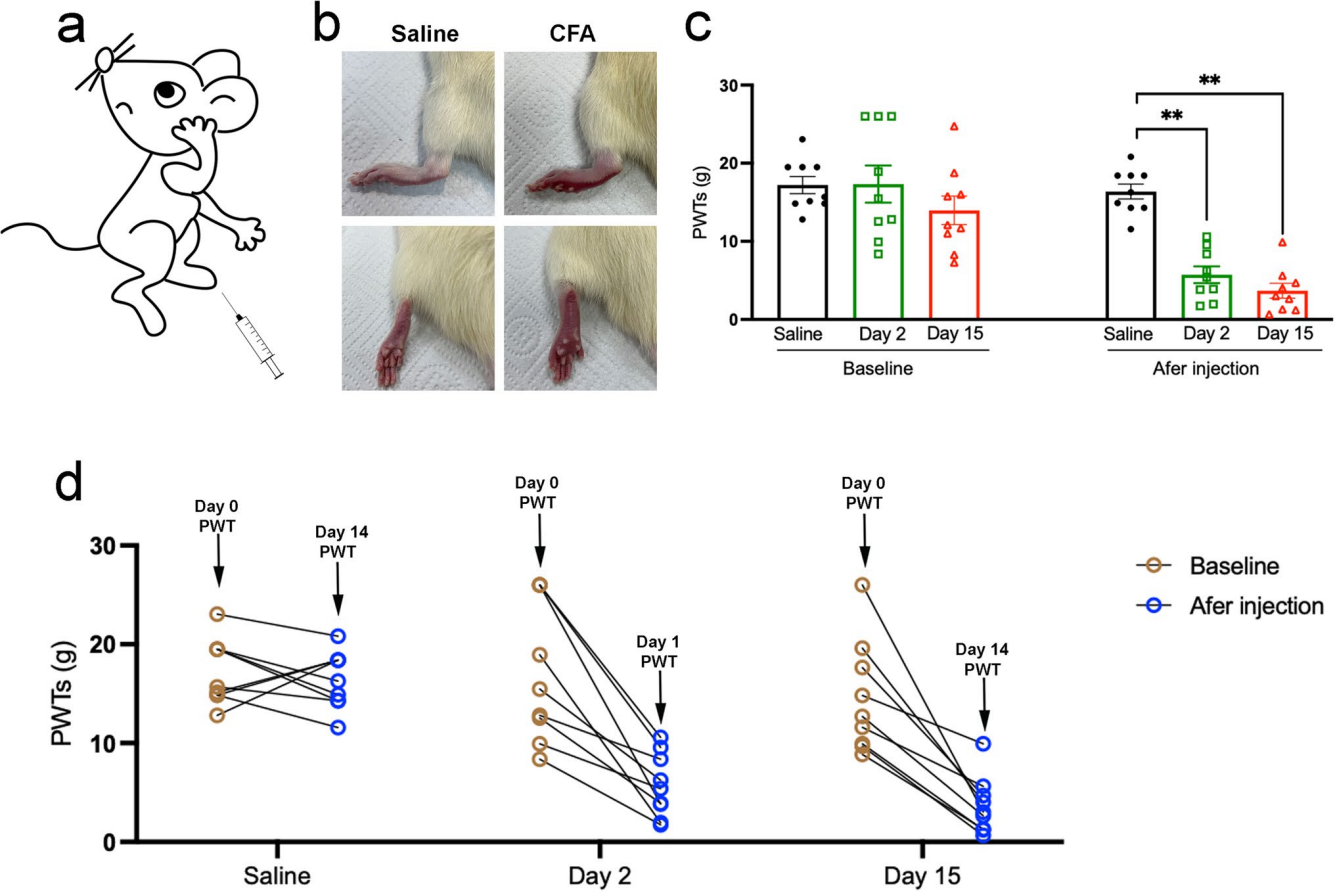
CFA injection induced mechanical hypersensitivity. **a** Inflammatory pain was induced with an injection of CFA in the plantar surface of the left hind paw. **b** CFA injection, but not saline, induced redness and swelling of the left hind paw. **c** The PWT baselines were similar in the three groups before CFA administration and significantly declined in the day 2 and day 15 groups after the CFA injection. **d** Compared with saline administration, the PWT of the day 2 and day 15 groups experienced a downward trend after CFA administration on day 0. All data represent the mean ± SEM, *n* = 9. **p < 0.01, compared to the saline group. CFA, complete Freund’s adjuvant; PWT, paw withdrawal threshold; SEM, standard error of the mean

We used a well-accepted CFA-induced chronic inflammatory pain rat model (Fig. 2a). As observed, the CFA injection induced redness and swelling of the left hind paw, while no obvious changes were observed in saline-treated rats (Fig. 2b). Mechanical allodynia was assessed using von Frey monofilaments. Baselines to punctuate mechanical stimuli were similar in the three groups before CFA administration. Mechanical hypersensitivity was evidenced from day 1 to day 14 by a significant decrease in PWT of the ipsilateral hind paw of CFA-injected animals compared to that of saline-treated rats (Fig. 2c, d).

### CFA Injection Induced Affective Response

**Fig. 3:**
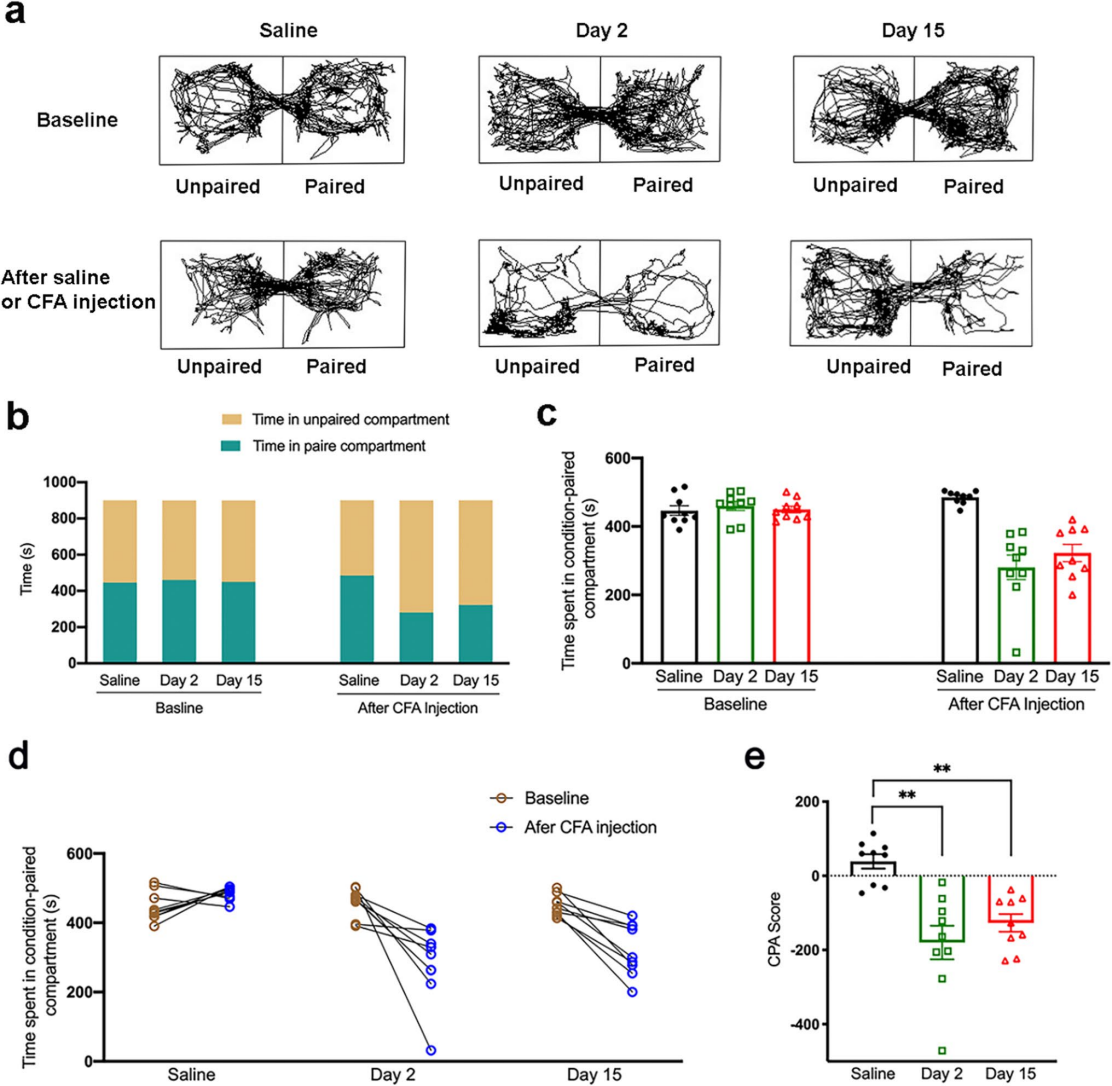
The effect of EA on pain aversion in CFA rats. **a** Trajectory chart of rats in the preconditioning and postconditioning test treated with saline and CFA induction. **b** The time structure in the C-CPA test. **c** The time spent in the condition-paired compartment in the three groups-saline, day 2, day 15 groups after the saline/CFA injection on day 0. **d** The trend of the time spent in the condition-paired compartment before and after the saline/CFA injection on day 0. **e** The CPA score was determined by subtracting the time spent in the pain-paired compartment during the postconditioning test. All data represent the mean ± SEM, *n* = 9. **p < 0.01, compared to the saline group. C-CPA, CFA-induced conditioned place aversion; CFA, complete Freund’s adjuvant; SEM, standard error of the mean

The C-CPA test was performed as described in previous research using Plexiglas place conditioning equipment [22]. The trajectory chart of the rats was recorded (Fig. 3a), and the time in the paired and unpaired compartments was quantified using the SMART 3.0 system (Fig. 3b). During the preconditioning period, a similar amount of time between the two compartments was observed for rats in saline, day 2, and day 15 groups, indicating no preference for a particular compartment. Nevertheless, after the hind-paw CFA injection was paired with a specific compartment, the rats spent less time in that compartment during the postconditioning test in comparison to that during the preconditioning test in both day 2 and day 15 groups (Fig. 3c). The data showed an aversion to the CFA-paired compartment over a period of 2 and 15 days. On the contrary, rats in saline group spent similar amounts of time in both compartments during both postconditioning and preconditioning tests, showing no aversion to the paired compartment (Fig. 3d). The CPA score indicated that the device chambers were neutral stimuli to the test rats and suggested that the CFA injection brought about pain-induced place aversion compared to the saline injection (Fig. 3e).

### Quantitative iTRAQ-Based Proteomic Analysis of the Amygdala in Pain Aversion Rats

#### Proteomic Data Revealed the Complexity of Pain Aversion

An abundant subfraction was obtained by a combination of Triton solubilisation and fractionation to study protein expression in the amygdala of rats with pain-aversion. Differential isotopic labelling with iTRAQ reagent was used to quantify differences in protein levels. A total of 6319 proteins were identified and quantitated (*FDR* < 0.01) in the three groups.

By a weighted average of all identified peptides distributed in all the given proteins, the alteration in the relative protein concentration in day 2 and day 15 groups compared to that of saline group was obtained from the iTRAQ 8-plex reporter ion ratios. The iTRAQ reporter ratios from 1.2 to 0.83 were determined as the threshold values for protein variation (Table 2).

**Table 2:** The amount of differentially expressed proteins (DEPs)

Comparisons	Upregulated	Downregulated	All
Day 2 vs saline	51	48	99
Day 15 vs saline	23	99	122

#### Analysis of Differentially Expressed Proteins in the Day 2 Group

**Fig. 4:**
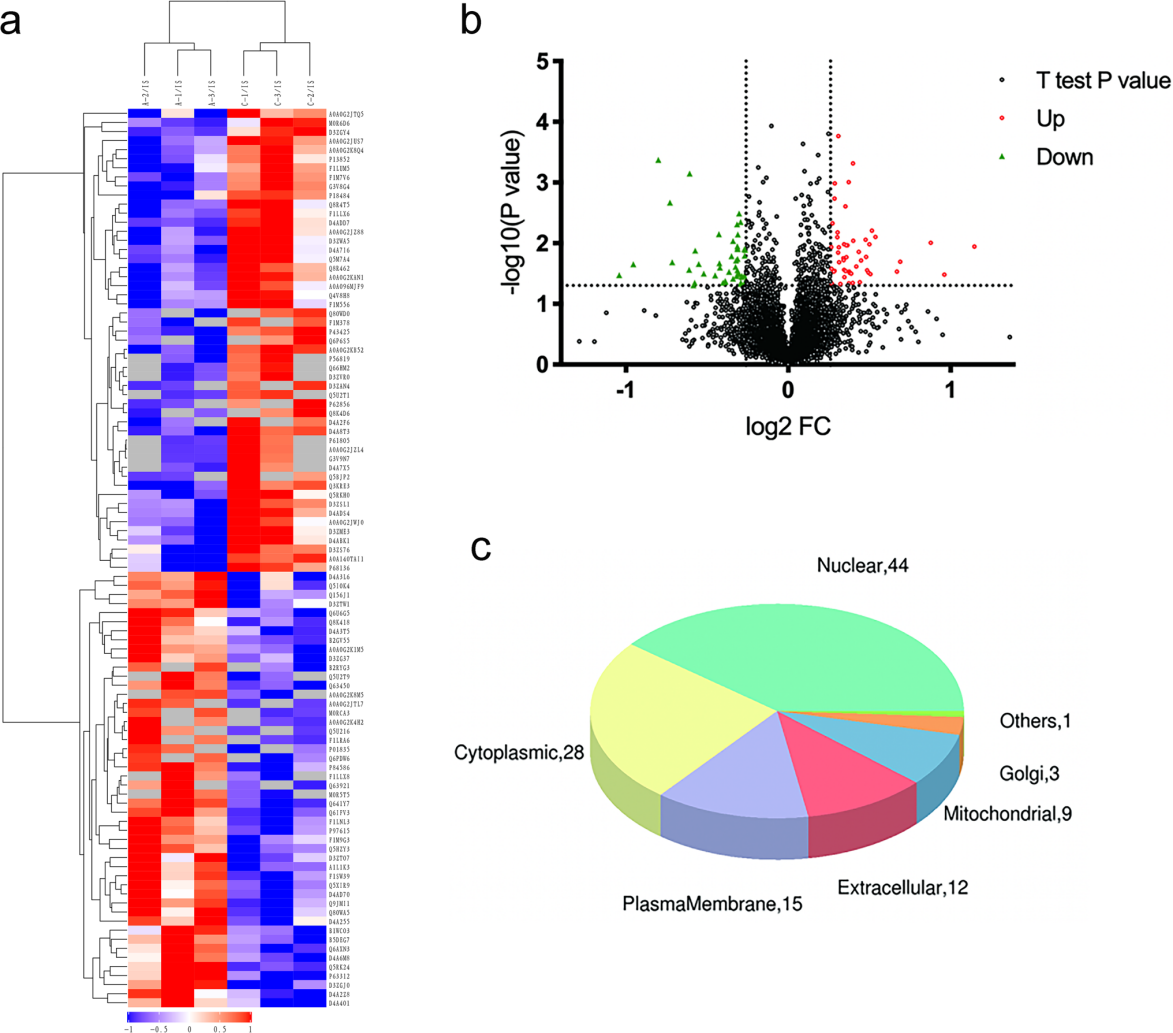
DEPs between the day 2 group and the saline group. **a** Clustering analysis is shown by a heatmap of DEPs. **b** Volcano plots exhibit significant DEPs. **c** Distribution of subcellular structure localisation of DEPs. DEP, differentially expressed proteins

Ninety-nine apparently altered proteins (either upregulated or lowered) were identified in the day 2 group compared to those in the saline group, which had at least one ratio with a *p*-value < 0.05. Heatmaps and hierarchical clustering (Fig. 4a) represent the functional analysis and ratio levels of all differentially expressed proteins (DEPs) in the amygdalae of rats in the day 2 group compared to those in the saline group using the hierarchical clustering algorithm, which was based on Euclidean distance. As a result, 99 DEPs (ESM Table 1) were identified, among which 51 were upregulated and 48 were downregulated in the day 2 group compared to the saline group (Fig. 4b). The subcellular structural localisation of DEP was predicted and classified statistically, and the subcellular structural changes were mainly reflected in the nucleus, cytoplasm, and plasma membrane (Fig. 4c).

**Fig. 5:**
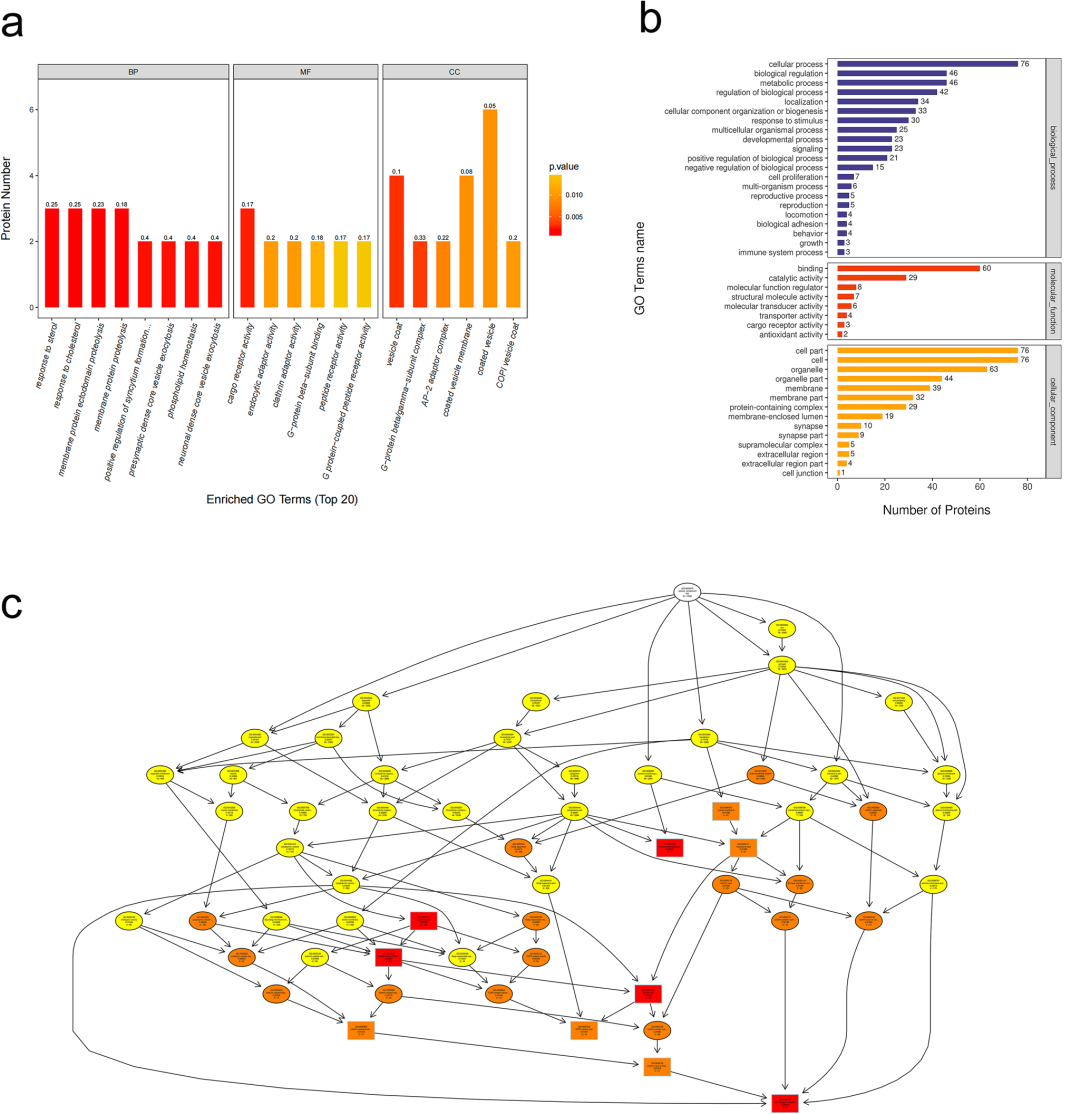
GO analyses of DEPs between the day 2 group and the saline group. **a** The top 20 GO enrichment terms. **b** GO annotation of level 2 terms. **c** CC directed acyclic graph (DAG) of top GO terms. DEP, differentially expressed proteins

We conducted GO analysis of the 99 DEPs in order to identify gene/protein functions and localizations. The identified proteins were classified into CC, MF, and BP. Moreover, they were estimated through GO annotations in the three larval stages, and the GO functional classification is illustrated in Fig. 5a.

GO annotation of the target protein set allowed for the classification of these proteins according to BP, MF, and CC. The proportion of proteins in each category may implied the impact of the three developmental stages on each GO category. Go annotation was used to analyse the DEPs, and statistical analysis of the considerably enriched GO terms from the data of the three developmental stages is exhibited in Fig. 5b.

The results indicated that the most notably enriched GO terms were CC, biological regulation, metabolic processes, and cellular processes. For biological processes, the DEPs were particularly abundant in neuronal and presynaptic dense-core vesicle exocytosis (Fig. 5c).

**Fig. 6:**
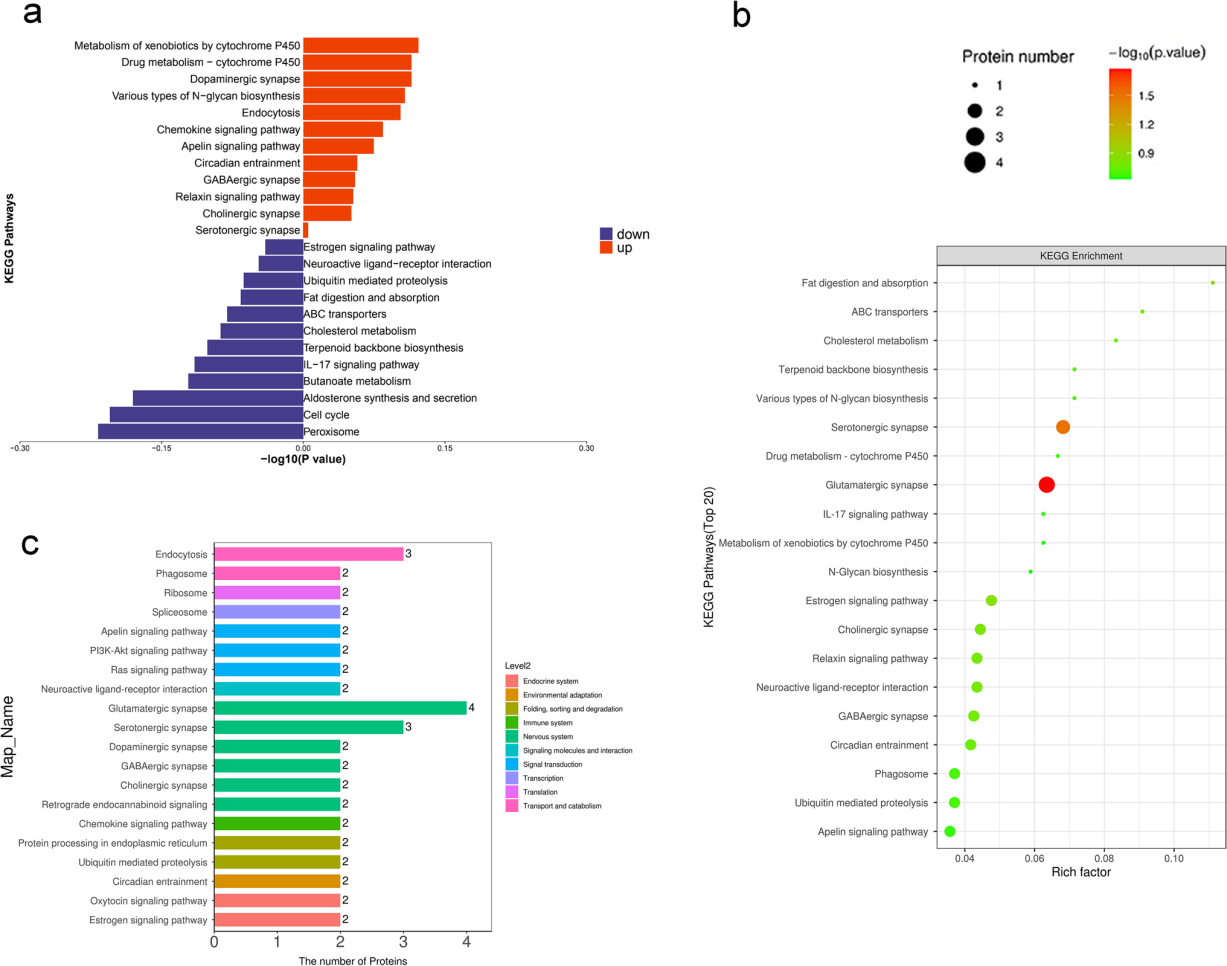
KEGG analyses of DEPs between the day 2 group and the saline group. **a** The top 20 most-enriched KEGG pathways. **b** Specific statistics for the top 20 most-enriched KEGG pathways; horizontal coordinate is the number of proteins; vertical coordinate shows the KEGG pathways. **c** Scatter plot of top 20 enriched KEGG pathways; horizontal coordinate is the rich factor; vertical coordinate shows the KEGG pathways; node size indicates how enriched KEGG pathways is; node colour indicates -log10 (*p*-value). DEP, differentially expressed proteins; KEGG, Kyoto Encyclopaedia of Genes and Genomes

The KEGG pathway was applied to estimate the level of importance of each protein enriched in the pathways, thereby determining proteins that have a significant impact on metabolism and signal transduction. KEGG was used to analyse the DEPs. Statistical assessments of the significantly enriched KEGG pathways are shown in Fig. 6a. The results showed that the most remarkably enriched KEGG pathways were those of xenobiotic metabolism, including that by cytochrome P450, peroxisomes, and dopaminergic synapses. The relationship between the KEGG pathway and the number of protein sequences is demonstrated in Fig. 6b and c. The results showed that the nervous system was the most annotated pathway for glutamatergic and serotonergic synapses.

#### Analysis of Differentially Expressed Proteins in the Day 15 Group

**Fig. 7:**
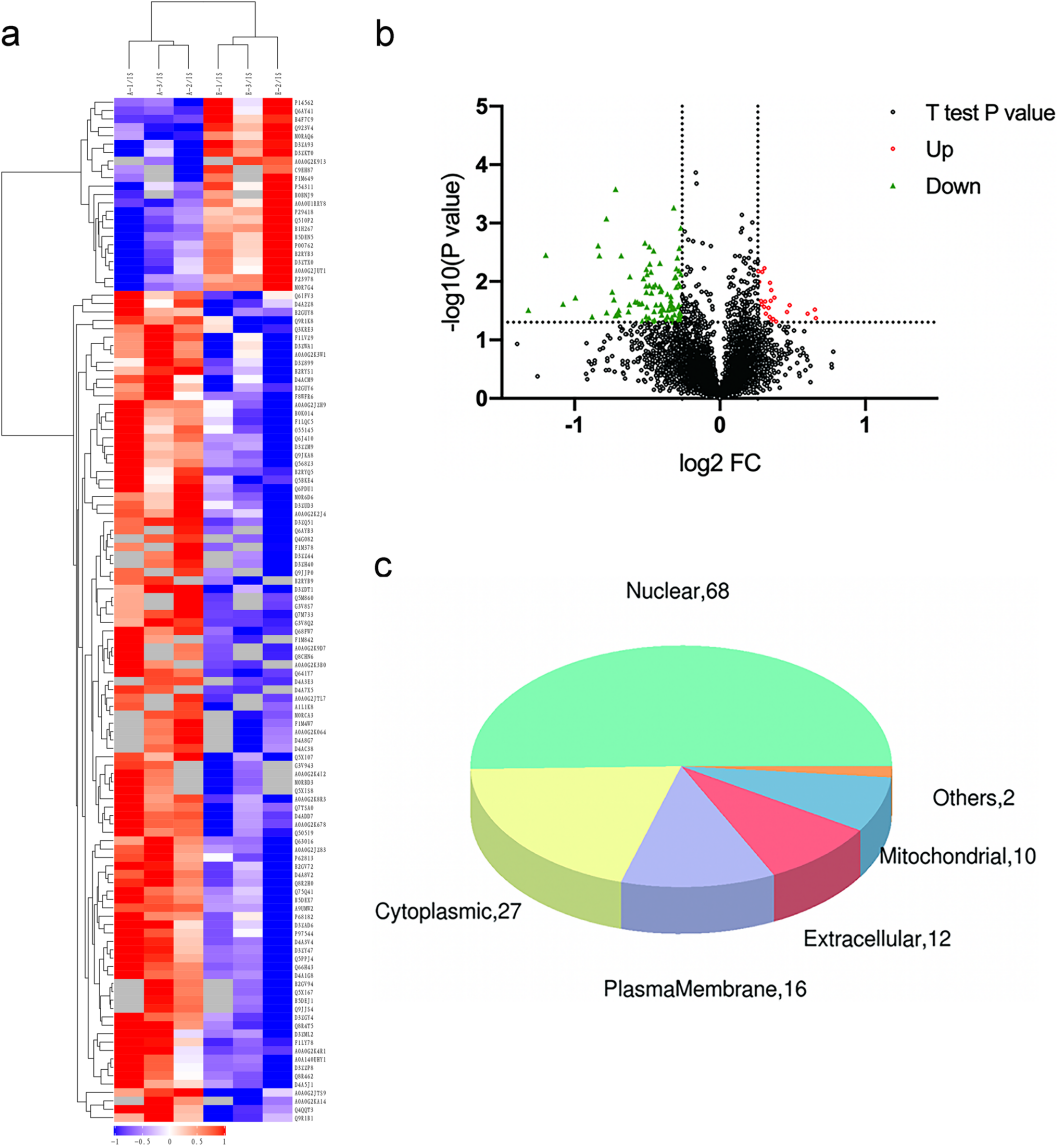
DEPs between the day 15 group and the saline group. **a** Heatmap view of the cluster analysis depicting the expression pattern of DEPs. **b** Volcano plots exhibit significant DEPs. **c** Distribution of subcellular structure localisation of DEPs. DEP, differentially expressed proteins

Meanwhile, 122 considerably altered proteins were evaluated in the pain aversion rats in the day 15 group and compared to those in the saline group (ESM Table 2). Heatmaps and hierarchical clustering (Fig. 7a) represent the functional analysis and ratio levels of all DEPs in the amygdalae of the day 15 group compared with those of the saline group using the hierarchical clustering algorithm, which was based on Euclidean distance. Of these, 24 proteins were increased and 99 proteins were decreased in the amygdalae of day 15 pain-averse rats (Fig. 7b). The subcellular structural localisation of DEP was predicted and classified statistically, with subcellular structural changes mainly in the nucleus, cytoplasm, and plasma membrane (Fig. 7c).

**Fig. 8:**
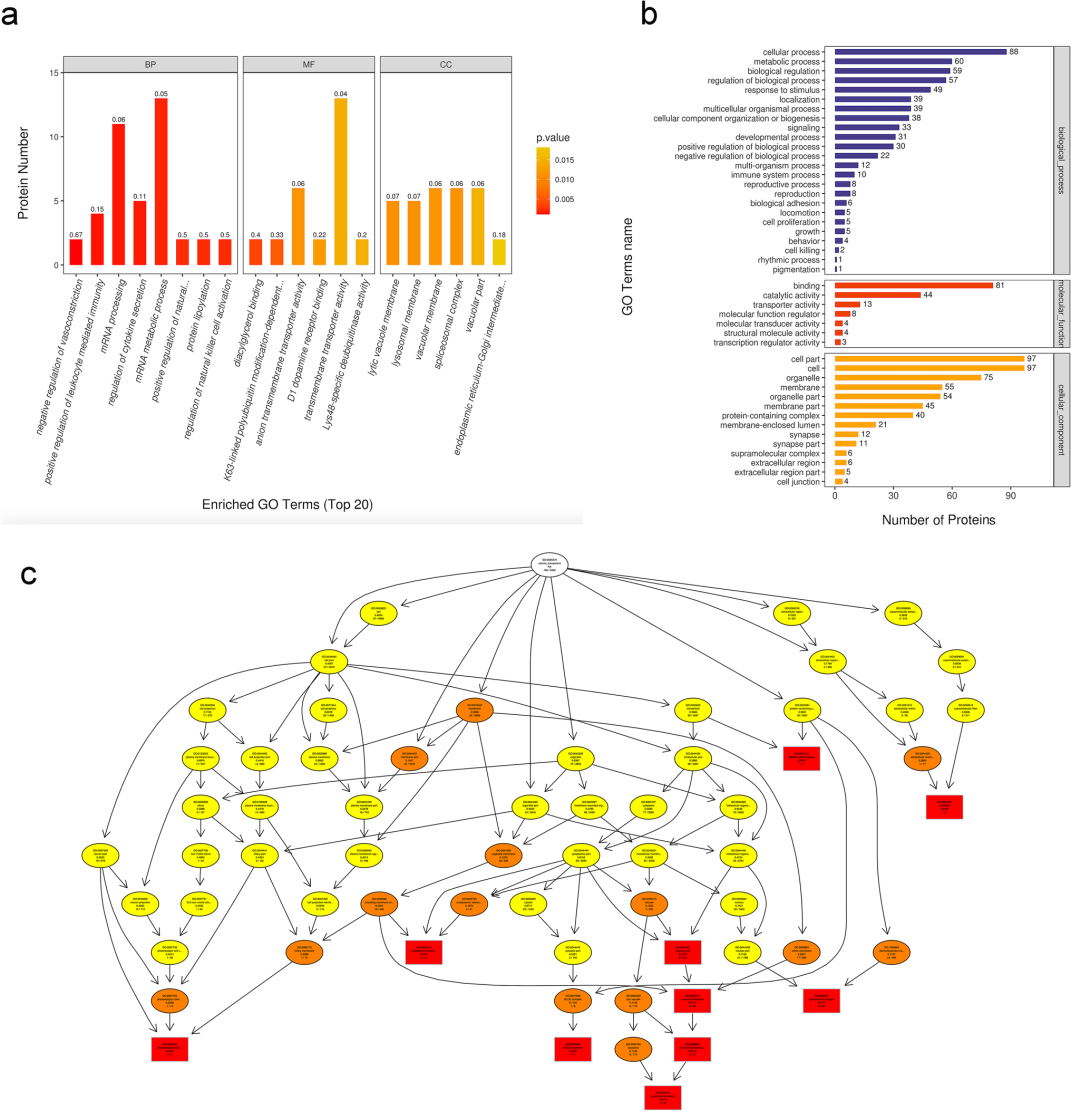
GO analyses of DEPs between the day 15 group and the saline group. **a** The top 20 GO enrichment terms. **b** GO annotation of level 2 terms. **c** CC directed acyclic graph (DAG) of top GO terms. DEP, differentially expressed proteins

We then conducted GO analysis for 123 DEPs, which were classified into CC, BP, and MF. Moreover, they were analysed by GO annotations in the three larval stages, and the GO functional classification is shown in Fig. 8a. The DEPs were analysed by GO annotation, and the statistical analysis of the GO terms significantly enriched from the data of the three developmental stages is shown in Fig. 8b. The results demonstrated that the most apparently enriched GO terms were CC, cellular processes, biological regulation, and metabolic processes. According to molecular function, DEPs were particularly enriched in dopamine receptor binding, anion transmembrane transporter activity, and K63-linked polyubiquitin modification-dependent protein binding (Fig. 8c).

**Fig. 9:**
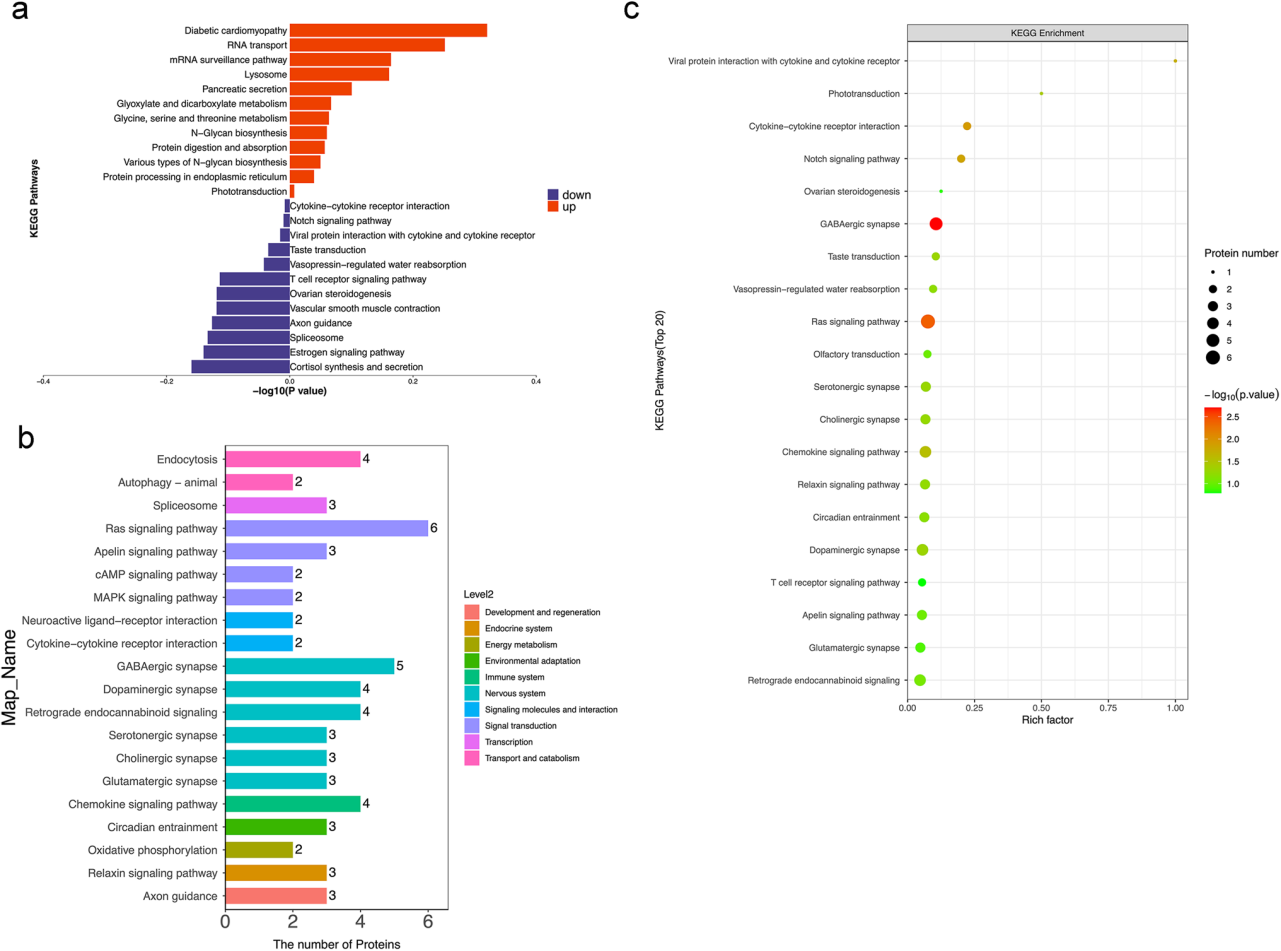
KEGG analyses of DEPs between the day 15 group and the saline group. **a** The top 20 most-enriched KEGG pathways. **b** Specific statistics for the top 20 most-enriched KEGG pathways; horizontal coordinate is the number of proteins; vertical coordinate shows the KEGG pathway. (**c**) Scatter plot of top 20 enriched KEGG pathways; horizontal coordinate is the rich factor; vertical coordinate shows the KEGG pathway; node size indicates how enriched KEGG pathways is; node colour indicates -log10 (*p*-value). DEP, differentially expressed proteins; KEGG, Kyoto Encyclopaedia of Genes and Genomes

Statistical assessment of the significantly enriched KEGG pathway by KEGG analysis of DEP is shown in Fig. 9a. The results showed that the most remarkably enriched KEGG pathways were diabetic cardiomyopathy, RNA transport, cortisol synthesis, and secretion. The relationship between the KEGG pathway and the number of protein sequences is shown in Fig. 6b and c. The results showed that the nerve system was the most annotated pathway to the glutamatergic, GABAergic, and serotonergic synapses.

#### Analysis of Differentially Expressed Proteins in the Saline, Day 2, and Day 15 Groups

**Fig. 10:**
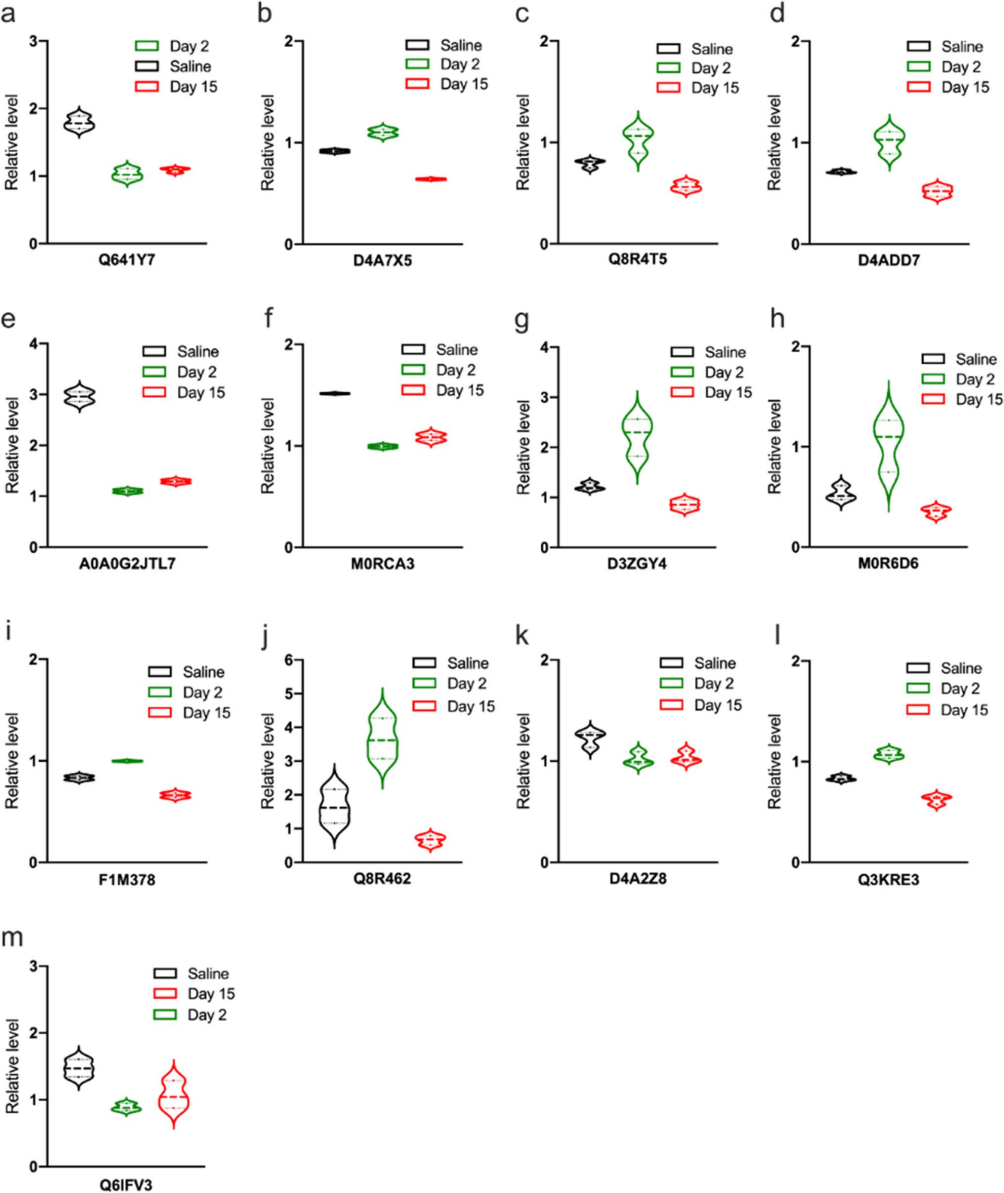
13 DEPs in both the day 2 and day 15 groups. **a** Q641Y7: 8-oxo-dGDP phosphatase NUDT18. **b** D4A7X5: protein Ppm1k. **c** Q8R4T5: general receptor for phosphoinositides. **d** D4ADD7: glutaredoxin 5 homologue. **e** A0A0G2JTL7: protein Ankib1. **f** M0RCA3: protein Zfpl1. **g** D3ZGY4: glyceraldehyde-3-phosphate dehydrogenase. **h** M0R6D6: uncharacterised protein. **i** F1M378: protein unc-13 homologue A. **j** Q8R462: glutamate transporter. **k** D4A2Z8: DEAH (Asp-Glu-Ala-His) box polypeptide 36. **l** Q3KRE3: guanine nucleotide-binding protein subunit gamma. **m** Q6IFV3: Keratin, type I cytoskeletal 15. DEP, differentially expressed proteins

Regarding the key proteins participating in pain aversion modulation, 13 proteins were found to differ between the day 2 and day 15 groups (Table 3), including Q8R64 and M0R6D6. The violin plots in Fig. 10 indicate the exact variation in the expression of each DEP. Among the 13 DEPs, Q8R64 denotes a glutamate transporter-1a (GLT1a), which utilises synaptic glutamate to maintain optimal extracellular glutamic levels, thereby preventing accumulation in the synaptic cleft and consequent excitotoxicity (Fig. 10j).

**Table 3:** Description of DEPs

Protein_ID	Description	Definition	Function	Day2/saline	Day15/saline
Q641Y7	8-oxo-dGDP phosphatase NUDT18			0.574389373	0.608172558
D4A7X5	Protein Ppm1k			1.202828351	0.699477037
Q8R4T5	General receptor for phosphoinositide 1-associated scaffold protein			1.299988943	0.714486298
D4ADD7	Glutaredoxin 5 homologue (S. cerevisiae)			1.413717159	0.728687695
A0A0G2JTL7	Protein Ankib1			0.371929128	0.436264122
M0RCA3	Protein Zfpl1			0.65654354	0.715790973
D3ZGY4	Glyceraldehyde-3-phosphate dehydrogenase	Glyceraldehyde 3-phosphate dehydrogenase	Carbon metabolism Alzheimer’s disease Biosynthesis of amino acids HIF-1 signalling pathway Glycolysis/Gluconeogenesis	1.837826416	0.704589896
M0R6D6	Uncharacterised protein	Cofilin	Pertussis Axon guidance Fc gamma R-mediated phagocytosis Regulation of actin cytoskeleton	1.94767651	0.667735825
F1M378	Protein unc-13 homologue A	Protein unc-13 A/B/C	Synaptic vesicle cycle	1.265864023	0.737587245
Q8R462	Glutamate transporter splice variant GLT1a	Solute carrier family 1 (glial high affinity glutamate transporter), member 2	Amyotrophic lateral sclerosis (ALS) Glutamatergic synapse	2.214674084	0.401431656
D4A2Z8	DEAH (Asp-Glu-Ala-His) box polypeptide 36	ATP-dependent RNA helicase DHX36	RNA degradation	0.809824354	0.805031089
Q3KRE3	Guanine nucleotide-binding protein subunit gamma	guanine nucleotide-binding protein G(I)/G(S)/G(O) subunit gamma-10	Cholinergic synapse Morphine addiction PI3K-Akt signalling pathway Relaxin signalling pathway Circadian entrainment GABAergic synapse Alcoholism Apelin signalling pathway Retrograde endocannabinoid signalling Chemokine signalling pathway Dopaminergic synapse Glutamatergic synapse Pathways in cancer Ras signalling pathway Serotonergic synapse Kaposi’s sarcoma-associated herpesvirus infection	1.294581532	0.795495921
Q6IFV3	Keratin, type I cytoskeletal 15	type I keratin, acidic	Staphylococcus aureus infection	0.603158306	0.726730723

#### The Organisation and Patterns of GLT-1 Immunoreactive Cells in the Amygdala

**Fig. 11:**
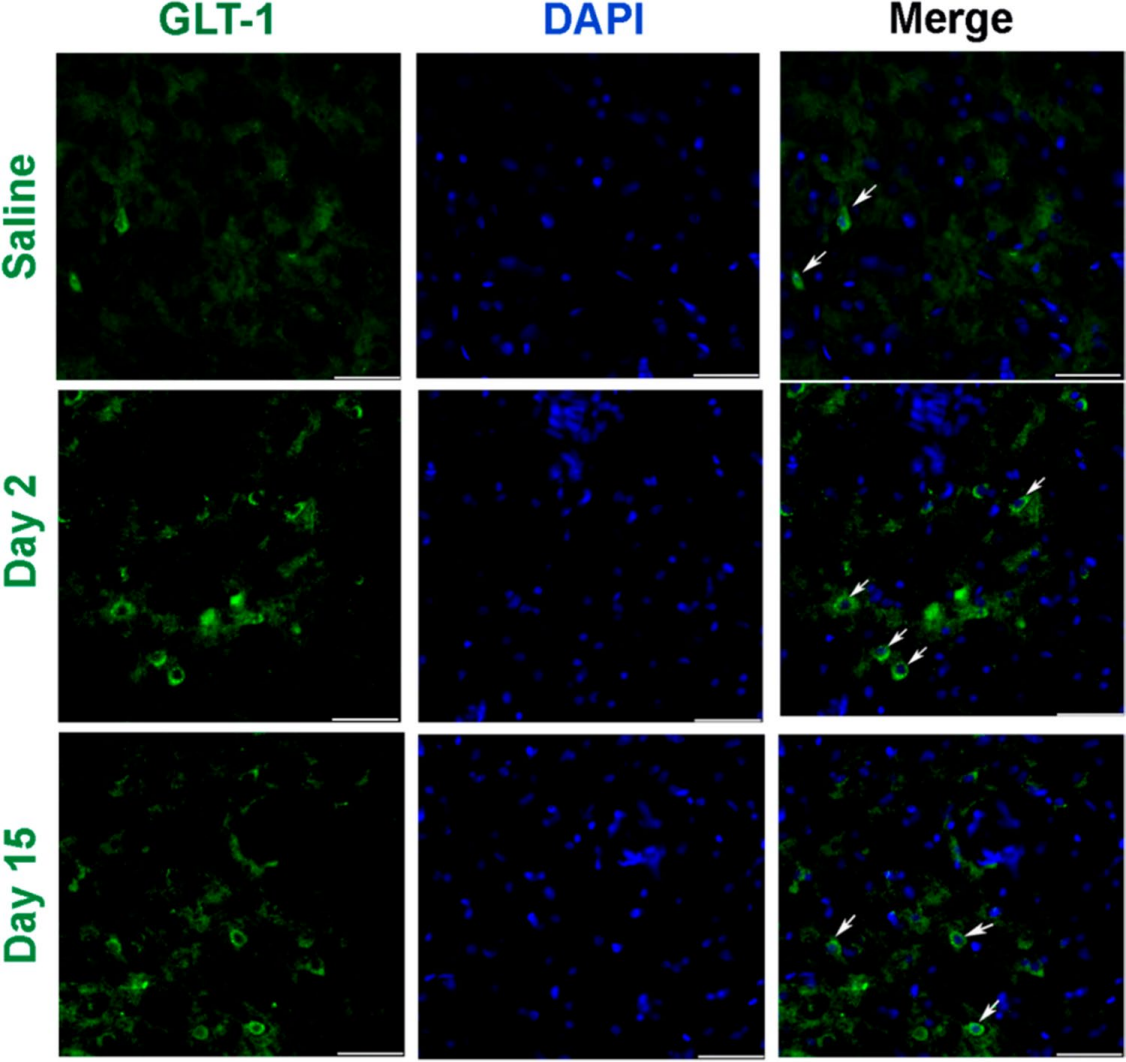
Representative immunofluorescence images showing an increase in the number of GLT-1-positive cells following CFA administration. Scale bar: 50 μm. **a** Representative immunofluorescence images in the saline group. **b** Representative immunofluorescence images in the day 2 group. **c** Representative immunofluorescence images in the day 15 group. CFA, complete Freund’s adjuvant; GLT-1, glutamate transporter-1

To investigate the organisation and patterns of GLT-1 immunoreactive cells in the amygdala, coronal sections of the amygdala region were subjected to immunohistochemistry with an anti-GLT-1 antibody. Brain sections were obtained from the bregma at − 1.23 mm, − 1.31 mm, and − 1.43 mm, respectively. We found that the GLT-1 signal on day 2 was intense, showing that GLT-1 increased during the early stage of pain aversion. Nevertheless, a lower expression of GLT-1 was observed in the day 15 group, which implied a reversal of GLT-1 expression in the later stage of pain aversion (Fig. 11).

## Discussion

In our study, the intraplantar CFA injection stimulated a rapid, regional inflammatory reaction, such as redness, swelling, and persistent pain. The inflammatory pain lasted for at least 14 days. Moreover, it caused an affective response to pain, as shown by the avoidance response evaluated by CPA.

The amygdala has emerged as a brain region relevant in the emotional-affective dimension of pain and pain modulation [24–28]. A wide range of neuropeptides identified in the amygdala have been explored for their roles in pain or affective modulation [29, 30]. Among them, the release of noradrenaline and the activation of α2 receptors in the amygdala have shown to be involved in stress-induced analgesia [31]. In addition, mechanical sensitivity significantly decreased 6 h post-induction in the formalin test in calcitonin gene-related peptide knockout mice. Also, neuropeptide S expression showed a time-dependent decrease over 3 weeks in a neuropathic pain model (spinal nerve ligation model) [32].

Concerning pain aversion, glutamate transmission in the basolateral part of the amygdala has shown to be involved in this response [33]. Moreover, the amygdala encodes aversive stimuli [34]. Endocannabinoids facilitate the extinction of aversive memories through selective inhibition of the GABAergic network in the amygdala [35]. Lastly, formalin-induced CPA and acetic acid-induced CPA can be eliminated by amygdala lesions [36, 37]. This study show that the amygdala may exert an influence on pain and pain aversion.

Nevertheless, the mechanistic underpinnings, specific cellular protein networks, and possible interactions within the major signalling pathways that govern these changes in the amygdala in chronic pain and pain aversion remain unclear. Innovative and unbiased approaches are consequently necessary to expand the current knowledge, identify new targets, and ultimately design new therapeutic options.

KEGG results showed that changes in glutamatergic, GABAergic, and serotonergic synapses occurred on day 2 and day 15, particularly glutamatergic ones. As mentioned, glutamate transmission in the basolateral amygdala exerts crucial effect on pain-induced aversion [33]. Moreover, long-term changes in glutamate synapses contribute to the expression of pain-induced aversive behaviour [38]. Metabotropic glutamate receptor 7 is involved in the neural processes subserving amygdala-dependent averse responses [39]. These suggest that glutamate synapses in the amygdala are related to pain aversion, but the exact mechanism remains elusive.

Therefore, we investigated protein expression in the amygdala on days 2 and 15 after CFA administration. On day 2, pain-associated behaviours were established, and aversive behaviours were fully induced by day 15. High throughput proteomics based on mass spectrometry is the core technique for large-scale protein identification [40]. Proteomics can elucidate the protein corresponding to a gene as well as identifying the structure and function of a particular protein [41]. Using unbiased proteome profiling, we identified 13 proteins that were altered in both day 2 and day 15 groups, including Q641Y7, D4A7X5, Q8R4T5, D4ADD7, A0A0G2JTL7, M0RCA3, D3ZGY4, M0R6D6, F1M378, Q8R462, D4A2Z8, Q3KRE3, and Q6IFV3 (Table 3).

These DEPs were involved in glutamatergic, cholinergic, and GABAergic synapses, morphine addiction, PI3K-Akt signalling pathway, as well as dopaminergic and serotonergic synapses. We used immunofluorescence to confirm the expression of DEPs. Among the 13 DEPs, Q8R462 was observed to increase its expression early after CPA injection stage and decline afterwards.

Q8R462 is a splice variant of the glutamate transporter GLT-1, which indicates the level of GLT-1. GLT-1 and their human homologues, excitatory amino acid transporter 2, and the Na^+^-dependent transmembrane symporters [42] take up synaptic glutamate to keep its optimal extracellular levels, thus preventing its accumulation in the synaptic cleft and subsequent excitotoxicity [43, 44]. Glutamate is the primary excitatory neurotransmitter in the central nervous system that initiates rapid signal transmission in the synapse before its re-uptake into the surrounding glia, specifically astrocytes. Most inputs to the amygdala involve excitatory pathways that use glutamate as a transmitter [45]. Glutamate is stored in the presynaptic vesicles. After glutamate is released from the presynaptic neuron, it binds to receptors on the post-synaptic neuron. This allows for the exchange of ions (Na + , K +) and the subsequent firing of action potentials. Glutamate then spills out of the synapse and is removed from the extracellular space by GLT-1. Within astrocytes, Glu can be metabolised in a variety of ways.

**Fig. 12:**
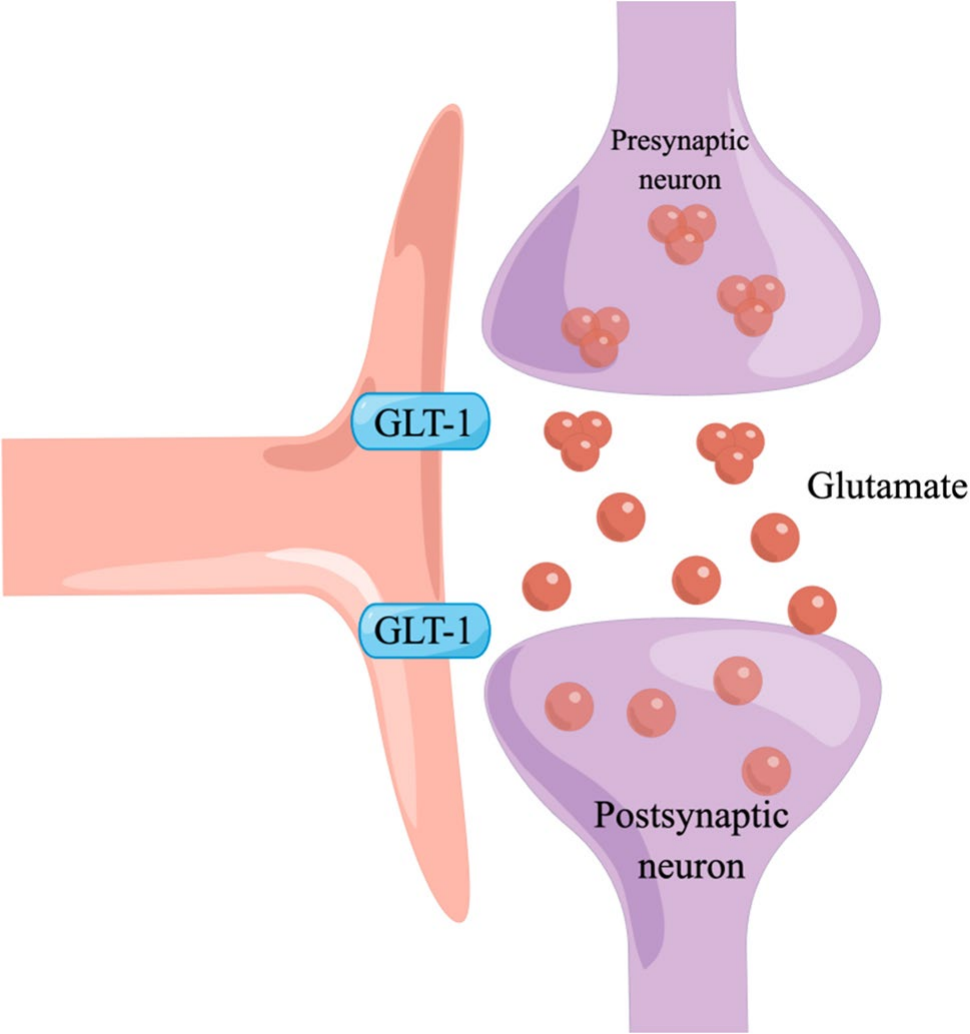
By figdraw. After glutamate is released from the presynaptic neuron, it binds to receptors on the postsynaptic neuron. This allows for the exchange of ions (Na + , K +) and subsequent firing of action potentials. Glutamate then spills out of the synapse and is cleared from the extracellular space by GLT-1. Within the astrocyte, glutamate can then be metabolised in a variety of ways. GLT-1, glutamate transporter-1

In our study, GLT-1 expression was upregulated on day 2 and downregulated on day 15. Pain aversion increased on day 2, and there was a downward trend on day 15. KEGG preliminary results showed that glutamate synapses associated with pain aversion changed significantly on days 2 and 15. Therefore, GLT-1 is highly likely to affect pain aversion through glutamate regulation, although the specific mechanism needs to be further elucidated (Fig. 12).

In summary, this study demonstrated that exposure to inflammatory pain resulted in pain-induced aversion, leading to extensive biological changes in the amygdala. Using proteomics analysis, 13 proteins were found to be different between the day 2 and day 15 groups, including Q8R64. Among the 13 DEPs, Q8R64 denotes GLT-1, which takes up synaptic glutamate to maintain optimal extracellular glutamic levels, thus preventing accumulation in the synaptic cleft and subsequent excitotoxicity. The temporal variation in GLT-1 expression was consistent with the variation tendency of pain aversion over time, which suggested a potential link between the modulation of pain aversion and the excitability of glutamatergic neurons.

### Supplementary Information

Below is the link to the electronic supplementary material.

Supplementary file1 (DOCX 53 KB)

## Data Availability

The datasets generated and/or analysed in the current study are available from the corresponding author.
