# Supplementary material for: Proteomic Analysis of the Amygdala Reveals Dynamic Changes in Glutamate Transporter-1 During Progression of Complete Freund’s Adjuvant-Induced Pain Aversion
Source: Mol Neurobiol. 2023 Aug 4;60(12):7166–84. doi: 10.1007/s12035-023-03415-7 (PMC10657795; doi:10.1007/s12035-023-03415-7)
Supplement: Supplementary file 1 — Supplementary file1 (DOCX 53 KB) [file 12035_2023_3415_MOESM1_ESM.docx]

Appendix Table 1. 99 significantly changed proteins in the Day 2 group

| **No.** | **Accession** | **description** | **Ratio(Day2/Saline)** | **t test *p* value** |
| --- | --- | --- | --- | --- |
| 1 | A0A0G2KB52 | Protein Map7 | 1.238609513 | 0.000171253 |
| 2 | D4A8T3 | Coatomer protein complex, subunit zeta 1 (Predicted) | 1.317892066 | 0.000484007 |
| 3 | Q3KRE3 | Guanine nucleotide-binding protein subunit gamma | 1.294581532 | 0.000983121 |
| 4 | P43425 | Guanine nucleotide-binding protein G(I)/G(S)/G(O) subunit gamma-7 | 1.219817916 | 0.001029802 |
| 5 | Q5U2T1 | Calfacilitin | 1.219024356 | 0.001839131 |
| 6 | D3ZAN4 | Protein Slc35b4 | 1.276933832 | 0.002474301 |
| 7 | G3V8G4 | Brevican core protein | 1.208097175 | 0.004714514 |
| 8 | A0A0G2KAN1 | Collagen alpha-2(I) chain | 1.429511973 | 0.006203965 |
| 9 | A0A140TAI1 | Ubiquilin 1, isoform CRA_a | 1.234273659 | 0.006636118 |
| 10 | A0A0G2JZL4 | Protein Nhsl1 | 1.451936764 | 0.00788694 |
| 11 | F1M7V6 | Cell adhesion molecule 4 | 1.233297001 | 0.007955813 |
| 12 | Q66HM2 | AP-2 complex subunit alpha-2 | 1.390913323 | 0.008784579 |
| 13 | P61805 | Dolichyl-diphosphooligosaccharide--protein glycosyltransferase subunit DAD1 | 1.319917647 | 0.009149653 |
| 14 | D3ZGY4 | Glyceraldehyde-3-phosphate dehydrogenase | 1.837826416 | 0.009834368 |
| 15 | D4ADD7 | Glutaredoxin 5 homolog (S. cerevisiae) (Predicted), isoform CRA_b | 1.413717159 | 0.01040245 |
| 16 | P68136 | Actin, alpha skeletal muscle | 1.269758 | 0.010477822 |
| 17 | D3ZSL1 | RNA binding protein fox-1 homolog | 1.287213873 | 0.01082246 |
| 18 | G3V9N7 | Protein Pacsin3 | 1.281958874 | 0.01086183 |
| 19 | Q8R462 | Glutamate transporter splice variant GLT1a (Fragment) | 2.214674084 | 0.01133942 |
| 20 | D4ADS4 | Protein Mgst3 | 1.202409667 | 0.011613944 |
| 21 | A0A0G2JUS7 | Transmembrane protein 11, mitochondrial | 1.24159461 | 0.011706996 |
| 22 | P56819 | Beta-secretase 1 | 1.351087093 | 0.013935497 |
| 23 | A0A0G2JZ88 | Protein 2900026A02Rik | 1.237280825 | 0.014280619 |
| 24 | D3ZVR0 | Gamma-tubulin complex component | 1.3940834 | 0.01689498 |
| 25 | A0A0G2K8Q4 | Phosphatase and actin regulator | 1.281855113 | 0.016989387 |
| 26 | F1M378 | Protein unc-13 homolog A | 1.265864023 | 0.017676143 |
| 27 | D4ABK1 | Protein Syngr3 | 1.266504943 | 0.01904553 |
| 28 | F1LLX6 | Calcium-dependent secretion activator 1 | 1.365009722 | 0.019301971 |
| 29 | Q80WD0 | Reticulon-4 receptor-like 1 | 1.61300372 | 0.02025802 |
| 30 | Q5RKH0 | Putative oxidoreductase GLYR1 | 1.243229383 | 0.020582687 |
| 31 | Q5M7A4 | Ubiquitin-like modifier-activating enzyme 5 | 1.336968921 | 0.024301815 |
| 32 | D4A7X5 | Protein Ppm1k | 1.202828351 | 0.02636747 |
| 33 | D3ZS76 | Protein Ttc9 | 1.270099755 | 0.027521891 |
| 34 | Q8K4D6 | Cytochrome P450 4X1 | 1.400584949 | 0.028035167 |
| 35 | D4A2F6 | Protein Nhlrc3 | 1.218809827 | 0.028555606 |
| 36 | A0A0G2JWJ0 | Protein phosphatase 1 regulatory subunit | 1.278536232 | 0.028889561 |
| 37 | D3ZME3 | Uncharacterized protein | 1.591006483 | 0.029300231 |
| 38 | D4A7I6 | Protein RGD1309995 | 1.214670783 | 0.029399562 |
| 39 | F1LUM5 | Protein Tubal3 | 1.297025705 | 0.029477807 |
| 40 | P62856 | 40S ribosomal protein S26 | 1.408256547 | 0.030481302 |
| 41 | Q8R4T5 | General receptor for phosphoinositides 1-associated scaffold protein | 1.299988943 | 0.030929915 |
| 42 | D3ZWA5 | Protein Kifap3 | 1.308451939 | 0.031702499 |
| 43 | P18484 | AP-2 complex subunit alpha-2 | 1.420310191 | 0.03227458 |
| 44 | M0R6D6 | Uncharacterized protein | 1.94767651 | 0.033141355 |
| 45 | Q5BJP2 | Spliceosome-associated protein CWC15 homolog | 1.271163326 | 0.036395048 |
| 46 | Q4V8H8 | EH domain-containing protein 2 | 1.357738222 | 0.044240284 |
| 47 | A0A096MJF9 | Protein Tmem132e | 1.317276042 | 0.045138606 |
| 48 | F1M556 | Protein Timm8a1 | 1.295988492 | 0.045605335 |
| 49 | Q6P6S5 | Tail-anchored protein insertion receptor WRB | 1.203745674 | 0.046045996 |
| 50 | P13852 | Major prion protein | 1.204715083 | 0.046236213 |
| 51 | A0A0G2JTQ5 | N-acetylgalactosamine kinase | 1.249544364 | 0.047215365 |
| 52 | Q641Y7 | 8-oxo-dGDP phosphatase NUDT18 | 0.574389373 | 0.000425196 |
| 53 | M0RCA3 | Protein Zfpl1 | 0.65654354 | 0.000710304 |
| 54 | Q6IFV3 | Keratin, type I cytoskeletal 15 | 0.603158306 | 0.002148513 |
| 55 | A0A0G2JTL7 | Protein Ankib1 | 0.371929128 | 0.002814452 |
| 56 | D3ZGJ0 | Protein Dhx32 | 0.810455378 | 0.003233416 |
| 57 | Q63450 | Calcium/calmodulin-dependent protein kinase type 1 | 0.816329294 | 0.004476198 |
| 58 | P84586 | RNA-binding motif protein, X chromosome retrogene-like | 0.804130513 | 0.004886424 |
| 59 | Q9JMI1 | Acetoacetyl-CoA synthetase | 0.743524313 | 0.00708425 |
| 60 | Q5RK24 | Phosphomevalonate kinase | 0.80677554 | 0.007184442 |
| 61 | P63312 | Thymosin beta-10 | 0.790374168 | 0.009198065 |
| 62 | F1LRA6 | Metabotropic glutamate receptor 8 | 0.804969472 | 0.011311661 |
| 63 | A1L1K3 | Anaphase-promoting complex subunit 5 | 0.80448021 | 0.012241531 |
| 64 | A0A0G2K8M5 | Protein Tmed4 | 0.829017957 | 0.012485614 |
| 65 | Q5HZY3 | Ubiquitin carboxyl-terminal hydrolase | 0.806355245 | 0.012557261 |
| 66 | F1SW39 | PC4 and SFRS1 interacting protein 1 | 0.831831756 | 0.012841013 |
| 67 | P97615 | Thioredoxin, mitochondrial | 0.672186934 | 0.013249111 |
| 68 | M0R5T5 | SH3 and multiple ankyrin repeat domains protein 2 | 0.830409874 | 0.016147342 |
| 69 | F1LNL3 | Protein Abca1 | 0.784609527 | 0.016372235 |
| 70 | A0A0G2K1M5 | Protein RGD1309079 | 0.805342431 | 0.018163816 |
| 71 | Q6AXN3 | Transmembrane emp24 domain-containing protein 5 | 0.800492692 | 0.019301998 |
| 72 | Q156J1 | Bcl-2-interacting death suppressor | 0.609500451 | 0.020684866 |
| 73 | Q6U6G5 | Zinc finger CCCH domain-containing protein 15 | 0.745145934 | 0.021368891 |
| 74 | B5DEG7 | Protein Champ1 | 0.682515397 | 0.022124384 |
| 75 | A0A0G2K4H2 | Protein Nsmaf | 0.516000469 | 0.022306407 |
| 76 | Q5U2T9 | FK506 binding protein 5 | 0.798919906 | 0.024437675 |
| 77 | P01835 | Ig kappa chain C region, B allele | 0.747081459 | 0.025233031 |
| 78 | Q5U216 | ATP-dependent RNA helicase DDX39A | 0.655291145 | 0.027430291 |
| 79 | Q5XIR9 | Ubiquitin-associated domain-containing protein 1 | 0.805030013 | 0.028067313 |
| 80 | Q80WA5 | Sodium myo-inositol transporter 1 | 0.776397312 | 0.029669381 |
| 81 | D4AD70 | Protein RGD1561636 | 0.810259925 | 0.031619386 |
| 82 | B2GV55 | Protein Ube2q1 | 0.69710249 | 0.031791225 |
| 83 | D4A2Z8 | DEAH (Asp-Glu-Ala-His) box polypeptide 36 (Predicted), isoform CRA_a | 0.809824354 | 0.032464923 |
| 84 | Q8K418 | Bombesin receptor subtype-3 | 0.485670649 | 0.033589613 |
| 85 | D4A6M8 | Melanoma antigen, family E, 2 (Predicted) | 0.731954359 | 0.033867869 |
| 86 | Q6PDW6 | 39S ribosomal protein L17, mitochondrial | 0.829531041 | 0.034289993 |
| 87 | B2RYG3 | Protein Vps9d1 | 0.802074891 | 0.034592619 |
| 88 | D3ZG37 | Protein Ppp6r1 | 0.820715446 | 0.03471064 |
| 89 | D4A3T5 | Protein C1ql3 | 0.819379121 | 0.036023817 |
| 90 | F1M9G3 | Opioid growth factor receptor-like protein 1 | 0.790165812 | 0.03856296 |
| 91 | D4A3L6 | ATP-binding cassette sub-family C member 8 | 0.764376147 | 0.042079648 |
| 92 | B1WC03 | Protein Aar2 | 0.832176513 | 0.042862854 |
| 93 | Q5I0K4 | Mrps9 protein (Fragment) | 0.764946949 | 0.04379785 |
| 94 | Q63921 | Prostaglandin G/H synthase 1 | 0.817096922 | 0.044316082 |
| 95 | D4A401 | Protein Tex10 | 0.75687566 | 0.045039446 |
| 96 | D3ZT07 | Platelet glycoprotein Ib beta chain | 0.670666592 | 0.045053828 |
| 97 | D4A255 | Protein Tldc1 | 0.81809314 | 0.046287889 |
| 98 | F1LLX8 | Lysosome-associated membrane glycoprotein 2 | 0.818609112 | 0.047291547 |
| 99 | D3ZTW1 | Protein Ints5 | 0.667329488 | 0.049608694 |

Appendix Table 2. 122 significantly changed proteins in the Day 15 group

| **No.** | **Accession** | **description** | **Ratio(Day15/Saline)** | **t test *p* value** |
| --- | --- | --- | --- | --- |
| 1 | D3ZA93 | Protein Acot13 | 1.235491199 | 0.005913838 |
| 2 | Q923V4 | F-box only protein 6 | 1.201352202 | 0.006690803 |
| 3 | B4F7C9 | Serine/threonine-protein kinase Chk1 | 1.223405177 | 0.006878334 |
| 4 | B1H267 | Sorting nexin-5 | 1.204918308 | 0.010165686 |
| 5 | M0R7G4 | MICOS complex subunit | 1.268834761 | 0.010536793 |
| 6 | D3ZKT0 | Phosphatidate cytidylyltransferase, mitochondrial | 1.276680412 | 0.014071715 |
| 7 | A0A0G2K9I3 | Golgin subfamily A member 4 | 1.296621808 | 0.018934041 |
| 8 | B5DEN5 | Eukaryotic translation elongation factor 1 beta 2 | 1.273743463 | 0.0215378 |
| 9 | B2RYB3 | Protein Srrm1 | 1.215606913 | 0.021580996 |
| 10 | Q6AY41 | Cell cycle control protein 50A | 1.241132049 | 0.022141526 |
| 11 | A0A0U1RRY8 | Protein Upf2 (Fragment) | 1.220379433 | 0.024836487 |
| 12 | M0RAQ6 | Hexokinase-1 | 1.393235794 | 0.025605369 |
| 13 | P23978 | Sodium- and chloride-dependent GABA transporter 1 | 1.23481784 | 0.027204488 |
| 14 | F1M649 | NLR family CARD domain-containing protein 4 | 1.261338279 | 0.028415858 |
| 15 | P00762 | Anionic trypsin-1 | 1.571617629 | 0.030151396 |
| 16 | P29418 | ATP synthase subunit epsilon, mitochondrial | 1.376760866 | 0.033491068 |
| 17 | Q5I0P2 | Glycine cleavage system H protein, mitochondrial | 1.518295078 | 0.03560704 |
| 18 | P14562 | Lysosome-associated membrane glycoprotein 1 | 1.244341591 | 0.035767971 |
| 19 | D3ZTX0 | Transmembrane emp24 domain-containing protein 7 | 1.267392901 | 0.04035568 |
| 20 | C9EH87 | Alpha II spectrin | 1.579971636 | 0.042210524 |
| 21 | B0BNJ9 | RCG44002, isoform CRA_a | 1.288959909 | 0.043960352 |
| 22 | P54311 | Guanine nucleotide-binding protein G(I)/G(S)/G(T) subunit beta-1 | 1.274618123 | 0.047911484 |
| 23 | A0A0G2JUT1 | Protein Cadm1 | 1.306656541 | 0.048325253 |
| 24 | Q641Y7 | 8-oxo-dGDP phosphatase NUDT18 | 0.608172558 | 0.000262905 |
| 25 | D4A3E3 | Protein Arid1a | 0.802856072 | 0.000541846 |
| 26 | D3ZQ51 | Protein Pak6 | 0.582335766 | 0.000838867 |
| 27 | Q68FW7 | Threonine--tRNA ligase, mitochondrial | 0.829539157 | 0.001203307 |
| 28 | D4A7X5 | Protein Ppm1k | 0.699477037 | 0.002195487 |
| 29 | Q7M733 | Hermansky-Pudlak syndrome 6 protein homolog | 0.559795395 | 0.002428646 |
| 30 | Q8R4T5 | General receptor for phosphoinositides 1-associated scaffold protein | 0.714486298 | 0.002516329 |
| 31 | G3V8Q2 | Alpha-internexin | 0.824127743 | 0.002682609 |
| 32 | D4ADD7 | Glutaredoxin 5 homolog (S. cerevisiae) (Predicted), isoform CRA_b | 0.728687695 | 0.002965386 |
| 33 | A0A0G2JTL7 | Protein Ankib1 | 0.436264122 | 0.003534419 |
| 34 | Q505I9 | Epsin 2 | 0.563011151 | 0.003603285 |
| 35 | Q66H43 | Uncharacterized protein C17orf59 homolog | 0.624587047 | 0.003612919 |
| 36 | A0A0G2K678 | YLP motif-containing protein 1 | 0.816833527 | 0.00393049 |
| 37 | Q8R2H0 | ATPase, H+ transporting, V1 subunit G isoform 2 | 0.751353115 | 0.004854228 |
| 38 | M0RCA3 | Protein Zfpl1 | 0.715790973 | 0.004988142 |
| 39 | D4A8V2 | Protein Ccdc177 | 0.813172915 | 0.006001918 |
| 40 | D3ZGY4 | Glyceraldehyde-3-phosphate dehydrogenase | 0.704589896 | 0.006148951 |
| 41 | Q4QQT3 | CUGBP Elav-like family member 1 | 0.823571009 | 0.006507582 |
| 42 | F1LY78 | Protein Dtx3 | 0.719477322 | 0.006814831 |
| 43 | A0A0G2K8R3 | Protein Lmo7 | 0.831104768 | 0.00736093 |
| 44 | D3ZDT1 | Protein Epb41l2 | 0.706411676 | 0.007568089 |
| 45 | Q5PPJ4 | Deoxyhypusine hydroxylase | 0.650845217 | 0.008187717 |
| 46 | D4A1G8 | Protein Cep170b | 0.810108294 | 0.00823843 |
| 47 | D4A3V4 | Protein Rnf214 | 0.806565004 | 0.00850347 |
| 48 | Q5XI07 | Lipoma-preferred partner homolog | 0.694509245 | 0.008832591 |
| 49 | Q7TSA0 | Mitochondrial Rho GTPase 2 | 0.821639468 | 0.010902314 |
| 50 | Q63016 | Large neutral amino acids transporter small subunit 1 | 0.73504452 | 0.011349283 |
| 51 | D3ZY47 | Protein RGD1559896 | 0.714294926 | 0.011658918 |
| 52 | A0A0G2JZ83 | Protein Agap3 | 0.703092506 | 0.011866411 |
| 53 | Q4G082 | Coq3 protein (Fragment) | 0.828082804 | 0.011981152 |
| 54 | A1L1K8 | Hyaluronan binding protein 4 | 0.74940042 | 0.012159939 |
| 55 | B2GV72 | Carbonyl reductase 3 | 0.72775824 | 0.012250744 |
| 56 | A0A0G2JTS9 | Protein Fbxo42 | 0.812217002 | 0.012464823 |
| 57 | A9UMW2 | Ndufa3 protein (Fragment) | 0.7565188 | 0.013847917 |
| 58 | A0A0G2K9D7 | Protein Pcdhgc3 | 0.754307758 | 0.014736356 |
| 59 | Q6AYB3 | Pre-mRNA-splicing factor ISY1 homolog | 0.598599348 | 0.015016148 |
| 60 | B2RYQ5 | Enhancer of rudimentary homolog | 0.704520214 | 0.015181535 |
| 61 | B2RYS1 | Neurensin 1 | 0.72182545 | 0.015664183 |
| 62 | Q6PDU1 | Serine/arginine-rich splicing factor 2 | 0.778828523 | 0.016420082 |
| 63 | Q8CHN6 | Sphingosine-1-phosphate lyase 1 | 0.791335994 | 0.017841849 |
| 64 | G3V943 | Protein Dnaaf5 | 0.501854716 | 0.018938482 |
| 65 | Q9JJS4 | Putative anion exchanger isoform 2 (Fragment) | 0.603257888 | 0.020455374 |
| 66 | B5DEX7 | Cation-transporting ATPase (Fragment) | 0.790965429 | 0.020960607 |
| 67 | M0R6D6 | Uncharacterized protein | 0.667735825 | 0.022123327 |
| 68 | B2GUY6 | Srrm2 protein (Fragment) | 0.675075714 | 0.022573578 |
| 69 | A0A0G2K4I2 | Protein Larp4b | 0.68983687 | 0.023642579 |
| 70 | F1LVZ9 | Protein Hectd3 | 0.790937892 | 0.023756683 |
| 71 | M0RBD3 | Protein Ksr2 | 0.681469241 | 0.024167379 |
| 72 | Q9JKA8 | Potassium/sodium hyperpolarization-activated cyclic nucleotide-gated channel 3 | 0.473243021 | 0.024238774 |
| 73 | Q568Z3 | Progestin and adipoQ receptor family member IV | 0.649584802 | 0.02465608 |
| 74 | Q6J4I0 | Protein phosphatase 1 regulatory subunit 1B | 0.754928583 | 0.02466147 |
| 75 | F1M378 | Protein unc-13 homolog A | 0.737587245 | 0.024981208 |
| 76 | B2GV94 | Fam134c protein | 0.794666264 | 0.025349133 |
| 77 | A0A0G2K4R1 | Protein Ppp1r12c | 0.822603252 | 0.025360888 |
| 78 | Q75Q41 | Mitochondrial import receptor subunit TOM22 homolog | 0.821107653 | 0.026080559 |
| 79 | Q5XI67 | F-box only protein 30 | 0.78736475 | 0.026193731 |
| 80 | B2RYB9 | LOC288526 protein | 0.832309089 | 0.026976557 |
| 81 | A0A0G2K3B0 | Growth factor receptor-bound protein 14 | 0.821943015 | 0.027052195 |
| 82 | F1M4W7 | Protein Cstf3 | 0.705890032 | 0.027141124 |
| 83 | D3ZAD6 | Neurexin-2 | 0.804000634 | 0.028507072 |
| 84 | Q9R1K8 | RAS guanyl-releasing protein 1 | 0.803676444 | 0.028737087 |
| 85 | Q5BKE4 | Sra1 protein | 0.758971087 | 0.029437068 |
| 86 | B2GUY8 | Protein Slc25a17 | 0.793721438 | 0.02974541 |
| 87 | P62813 | Gamma-aminobutyric acid receptor subunit alpha-1 | 0.764493133 | 0.03028438 |
| 88 | A0A0G2K3W1 | Protein Vwa8 | 0.780111715 | 0.030673898 |
| 89 | B5DEJ1 | SLIT-ROBO Rho GTPase-activating protein 2 | 0.617226122 | 0.030823075 |
| 90 | Q8R462 | Glutamate transporter splice variant GLT1a (Fragment) | 0.401431656 | 0.030838299 |
| 91 | F1LQC5 | Receptor protein serine/threonine kinase | 0.753198128 | 0.031791453 |
| 92 | D3ZZP8 | Protein Cul9 | 0.730362149 | 0.032291572 |
| 93 | A0A0G2K2J4 | RNA binding protein fox-1 homolog | 0.646393914 | 0.032680309 |
| 94 | Q9R1B1 | Mitochondrial import inner membrane translocase subunit Tim10 B | 0.582520687 | 0.033974931 |
| 95 | A0A0G2K064 | Tyrosine-protein phosphatase non-receptor type | 0.825034375 | 0.03403024 |
| 96 | D3ZH40 | Protein Otud7b | 0.612896668 | 0.035516789 |
| 97 | A0A0G2JZH9 | Protein Dhx57 | 0.83034252 | 0.03637965 |
| 98 | F8WFR6 | Glycogenin-1 | 0.617835182 | 0.036623291 |
| 99 | D4A2Z8 | DEAH (Asp-Glu-Ala-His) box polypeptide 36 (Predicted), isoform CRA_a | 0.805031089 | 0.036716925 |
| 100 | D4A5J1 | Protein Kbtbd11 | 0.75745963 | 0.036984036 |
| 101 | B0K014 | D-tyrosyl-tRNA(Tyr) deacylase | 0.824385747 | 0.037372487 |
| 102 | Q9JJP0 | Sodium-dependent phosphate transporter 1 | 0.608500595 | 0.038269714 |
| 103 | D3ZWA1 | Protein Fam63b | 0.777593579 | 0.03932882 |
| 104 | Q5XIS8 | Mitochondrial dynamics protein MID51 | 0.703557317 | 0.039482393 |
| 105 | D3ZZM9 | Protein Ccsap | 0.54361419 | 0.039923051 |
| 106 | A0A140UHY1 | Protein Ttc4 | 0.779230238 | 0.04015609 |
| 107 | D3Z899 | Protein Fam73b | 0.824698375 | 0.040365302 |
| 108 | Q3KRE3 | Guanine nucleotide-binding protein subunit gamma | 0.795495921 | 0.040932331 |
| 109 | P97544 | Phospholipid phosphatase 3 | 0.824683581 | 0.041469713 |
| 110 | D4AC38 | Protein Ago1 | 0.712956109 | 0.041847508 |
| 111 | D3ZML2 | Serine/threonine-protein kinase BRSK2 | 0.794971972 | 0.043364511 |
| 112 | D4A8G7 | Protein Snw1 | 0.826873621 | 0.044254048 |
| 113 | P68182 | cAMP-dependent protein kinase catalytic subunit beta | 0.773682286 | 0.044413749 |
| 114 | A0A0G2KA14 | Protein Clec16a | 0.816904612 | 0.045912166 |
| 115 | F1M842 | Protein Tp53bp1 | 0.732341056 | 0.04591947 |
| 116 | D4ACM9 | Protein Mfap1a | 0.690719198 | 0.047046507 |
| 117 | D3ZUD3 | Protein Wipf2 | 0.76733894 | 0.047288576 |
| 118 | Q6IFV3 | Keratin, type I cytoskeletal 15 | 0.726730723 | 0.04736671 |
| 119 | D3ZZ44 | Protein Elfn1 | 0.702813405 | 0.048841519 |
| 120 | G3V8S7 | Potassium intermediate/small conductance calcium-activated channel, subfamily N, member 3, isoform CRA_a | 0.825751615 | 0.049259529 |
| 121 | O55145 | Fractalkine | 0.801488003 | 0.049399119 |
| 122 | Q5M860 | Protein Arhgdib | 0.76989037 | 0.04955533 |
